# Selenium-Rich Ricegrass Juice Improves Antioxidant Properties and Nitric Oxide Inhibition in Macrophage Cells

**DOI:** 10.3390/antiox7040057

**Published:** 2018-04-13

**Authors:** Rattanamanee Chomchan, Panupong Puttarak, Adelheid Brantner, Sunisa Siripongvutikorn

**Affiliations:** 1 Interdisciplinary Graduate School of Nutraceutical and Functional Food, Prince of Songkla University, Hat-Yai, Songkla 90112, Thailand; pui_galz@hotmail.com; 2 Department of Pharmaceutical Sciences, Prince of Songkla University, Hat-Yai, Songkla 90112, Thailand; panupong.p@psu.ac.th; 3 Department of Pharmacognosy, Institute of Pharmaceutical Sciences, University of Graz, Graz 8010, Austria; adelheid.brantner@uni-graz.at; 4 Department of Food Technology, Faculty of Agro-Industry, Prince of Songkla University, Hat-Yai, Songkla 90112, Thailand

**Keywords:** selenium, ricegrass juice, antioxidant enzymes, lipid peroxidation, nitric oxide inhibition

## Abstract

Ricegrass juice (*Oryza sativa* L.) was introduced as a functional food as the consumption of sprouts or seedlings has been claimed to provide high nutritive value. Selenium (Se) is a trace mineral that plays a key role in the human antioxidation scheme. Supplementation of Se into plants is one strategy to enhance plant bioactivities, and the consumption of Se plant foods may confer superior health benefits. In this study, ricegrass juice extract was analyzed for its major phenolic components. The effect of ricegrass juice extracts bio-fortified with 0, 10 and 40 mg Se/L named as RG0, RG10, and RG40, respectively, were investigated for a percentage of cell viability, changes of endogenous antioxidant enzymes, lipid peroxidation, and nitric oxide inhibition in RAW264.7 macrophage cells. Flavone glycosides, namely chrysoeriol arabinosyl arabinoside derivatives, were found to be the foremost bioactive components in ricegrass juice extract indicated by UHPLC-MS. The results of cell culture assessment revealed that RG40 showed an ability to promote macrophage cell proliferation at low concentration. Ricegrass juice extract in all treatments possessed the ability to reduce malondialdehyde content, which may be regarded as the bioactivity of phenolic compounds. Moreover, Se also played a role in this effect since RG40 showed the greatest ability via increasing the level of GPx enzyme. It was also discovered that phenolic compounds in the extracts played a role in inhibiting nitric oxide in LPS-induced RAW264.7 cells. Furthermore, RG40 expressed significantly higher NO inhibition properties at IC_50_ 118.76 µg/mL compared to RG0 and RG10, at 147.02 and 147.73 µg/mL, respectively. Se bio-fortified ricegrass juice could be considered as a new potent functional food that can lower the risk of oxidative stress and chronic inflammation diseases.

## 1. Introduction

At present, new directions in human consumption consider daily nutrition to include essential nutrients and energy and bioactive compounds that demonstrate the additional benefits to human health [1]. Since plants at the beginning of their growing stage have been reported to contain large amounts of quality phytonutrients such as amino acids, trace elements, vitamins, and various phytochemicals to protect them from danger and prepare for expansion, sprouts or young plant of cereals, grains, or legumes are currently of interest as a new kind of vegetable [2]. Consequently, the consumption of sprouts in the usual diet may deliver many bioactive components and improve well-being. Wheatgrass juice, the eminent sprout juice squeezed from young leaves of wheat, has long been used as a health-promoting drink and has been the subject of health-supportive pharmacological studies. Ricegrass is a brand-new sprout which was recently introduced to substitute the use of wheatgrass, especially in high rice production areas as an economy ingredient. The use of ricegrass also arouses the utilization of low-cost variety rice seeds since it was proven to contain higher levels of polyphenol and possess comparable antioxidant activities to wheatgrass juice [3,4]. Additionally, many varieties of young ricegrass in Thailand had been earlier reported to inhibit lipid peroxidation and provide DNA protective properties against exposure to Fenton reaction due to numerous secondary metabolite components such as anthocyanin and phenolics [5].

Currently, a study on how to produce or improve the functional foods or ingredients quality is of interest since there are many methods available to boost the benefits of ingredients [6]. Minerals are the basic elements required for every unit of living cells. The procedure of mineral supplementation into plants is one of the outstanding methods to provide additional competence to ingredients [7]. It is an inexpensive technique which can provide mineral supplementation as well as possibly improve the biological properties of plants from the combined effects of phytochemical compounds and an increased content of minerals. Selenium (Se) is an essential trace mineral required by humans. It has a major function in the antioxidant system, and it is claimed to have outstanding efficiency among antioxidant minerals involved in many endogenously antioxidant enzymes [8]. Moreover, it has various roles in improving wellness as well as preventing disorder conditions or diseases, including boosting immunity, helping defense against cancer, anti-diabetic, regulation of thyroid function, anti-inflammation, and anti-aging [9]. Se has been supplemented with plentiful plants worldwide to improve plant nutritional values and exert advantageous effects on plant bioactivities [10]. Therefore, the supplementation of Se into ricegrass may possibly be a key to improving the typical ricegrass to be a novel functional food.

Humans currently face many harmful radicals stimulating in everyday life. Diet, stress and pollution are major factors that lead to oxidative stress and cause various health complications. To fight these damages, antioxidant protection is needed to prevent ROS related diseases [11]. Also, in response to injury or invaders, the immune system reacts by generating inflammation. Inflammatory cytokines like nitric oxide (NO) are released for host defense response. Under normal conditions, it plays a part in the regulation of vasodilatation and neurological issues. On the other hand, overproduction of NO induces tissue damage and associated with chronic inflammation [12]. As many of the synthetic antioxidants and anti-inflammation have been widely used, risk of toxicity should be of concern. Accordingly, growing attention has been paid to the development of efficient compounds from natural resources that can naturally modulate the antioxidant protection and inflammation responses. The objective of this study is to identify the type of phenolic compounds present and the Se content of ricegrass juice extract in relation to the supplementation of sodium selenite into ricegrass. The effect of ricegrass juice (RG0) and the Se bio-fortified ricegrass juice (RG10, RG40) was then investigated for a percentage of cell viability, antioxidant enzymes activity, lipid peroxidation, and the inhibition of nitric oxide in RAW264.7 murine macrophage cells.

## 2. Materials and Methods

### 2.1. Reagents

Reduced glutathione (GSH), 5,50-dithiobis-(2-nitrobenzoic acid) (DTNB), riboflavin, l-methionine, nitroblue tetrazolium chloride (NBT), bovine serum albumin (BSA), Coomassie brilliant blue G-250 dye, malondialdehyde (MDA), 2-thiobarbituric acid (TBA), formic acid, HPLC grade acetonitrile (ACN), HPLC grade methanol, lipopolysaccharide (LPS) from *E. coli*, l-nitro-arginine (LNA), and 3-(4,5-dimethylthiazol-2-yl)-2,5-diphenyltetrazolium bromide (MTT) were acquired from Sigma Aldrich Co. (St. Louis, MO, USA). Hydrogen peroxide (H_2_O_2_) and trichloroacetic acid (TCA) were purchased from Thermo Fisher Scientific Co. (San Jose, CA, USA). Reagents and media for cell line included trypan blue dye, trypsin-EDTA, fetal bovine serum (FBS), penicillin, streptomycin, and Roswell Park Memorial Institute (RPMI) 1640 medium were purchased from Gibco BRL, Life Technologies Inc. (Rockville, MD, USA) and Griess reagent was from Merck (Darmstadt, Germany).

### 2.2. Plantation and Plant Extract Preparation

Rice grains (*Oryza sativa* L. cv. Chainat 1), provided by the Phatthalung Rice Research Center, Thailand, were soaked in water for 24 h, drained and washed with distilled water. Soaked rice seeds were planted in trays and left in darkness for 48 h to allow the germination. Se solution was prepared by divided into 3 groups of supplementation containing (1) control (without Se) (2) 10 mg Se/L and (3) 40 mg Se/L using sodium selenite as Se source. Se solution was applied into germinated rice seeds at a ratio 1:1 *v/w*. Ricegrass was cultivated hydroponically without any supporting materials under natural light with a day/night average temperature of 33/25 °C, relative humidity 63% ± 5.0%, and photoperiod 12/12 (day/night). Leaves of rice plants or ricegrass at day 8 were harvested and extracted with water at a ratio of 1:2 (*w/v*) using a juicer (DA-900, Hurom Co., Ltd., Gimhae-si, Gyeongsangnam-do, South Korea). The samples were filtered and centrifuged at 10,000× *g* for 10 min. The supernatant was subjected to freeze-drying and the obtained plant powder extracts were stored at 4 °C in dark bottle. 

### 2.3. Selenium Content Determination

Total Se content of plant extracts was determined using an inductively coupled plasma optical electron spectrophotometer (ICP-OES). Approximate 0.1 g of extracts were digested with 3 mL of concentrated HNO_3_ and 1 mL of 30% H_2_O_2_ in a digestive stove heated at 180 °C for 1.5 h. The digested product was reconstituted to 10 mL with Milli-Q water and an auto-sampler for total Se content [13].

### 2.4. Total Polyphenol Content

The total extractable phenolic content of ricegrass juice extracts was measured using a method modified from Singleton and Rossi [14]. Briefly, 20 μL of the extract was added to 96-well microplate. Then, 100 μL of Folin reagent (10% *v/v*) and 80 μL of Na_2_CO_3_ (7.5% *w/v*) were added and mixed thoroughly. After incubation for 30 min in the dark at ambient temperature, the absorbance was measured at 765 nm using a microplate reader and expressed as mg pyrogallol equivalent (PYE)/g extract. 

### 2.5. Phenolic Profiles Identification Using UHPLC-DAD-ESI-MS

Main polyphenol compounds in ricegrass juice extracts were investigated using a Thermo Scientific (Dionex Softron GmbH., Germering, Germany) Ultimate 3000 Ultra High-Performance Liquid Chromatography (UHPLC) system equipped with Diode Array Absorbance Detector, Electron Spray Ionization, and LTQ XL mass detector (DAD-ESI-MS) The extracts of 10 mg/mL were dissolved in HPLC water and filtered with a sterile syringe filter of 0.45 µm. Separation was carried out using a Purosper STAR (250 mm × 4.6 mm) with LiChrocart, RP-18 column end-capped with 5 µm diameter particles (Merck, Darmstadt, Germany) as the stationary phase. The mobile phase consisted of H_2_O containing 0.5% formic acid (solvent A) and acetonitrile (solvent B), using the following gradient of solvent B: 0.00–10.00 min, 0–10.0%; 10.00–15.00 min, 10.0%; 15.00–20.00 min, 10.0–15.0%; 20.00–30.00 min, 15.0–25.0%; 30.00–35.00 min, 25.0%; 35.00–45.00 min; 25.0–100% followed by washing with 100% methanol and re-equilibration. The flow rate was 0.8 mL/min, and the column temperature was 40 °C with the injection volume of 20 μL. The MS parameters were as follows for both the negative and positive mode: heater temperature: 250 °C; capillary temperature: 330 °C; sheath gas flow: 50 arbitrary units; auxiliary gas flow: 10 arbitrary units. The mass data for the molecular ions were processed with Thermo XcaliburTM software version 2.2.44 (Thermo Scientific., Hemel Hempstead, UK).

### 2.6. Cell Culture Model

RAW264.7, mouse murine macrophage cells were purchased from the American Type Culture Collection (Manassas, VA, USA). Roswell Park Memorial Institute (RPMI) 1640 medium supplemented with 1.5% sodium bicarbonate, 10% fetal bovine serum (FBS), and 1% penicillin-streptomycin was used for the maintenance of cells at 37 °C, 5% CO_2_, in a fully humidified incubator. Phosphate buffer saline (PBS) at pH 7.2 was used to wash the cells and throughout the experiment.

### 2.7. Cell Viability Assay

The MTT assay was used to determine the percentage of cell viability. Cells grown at 80–90% confluence were harvested with 0.25% trypsin—EDTA and suspended in a fresh medium. Cell counts were determined using a standard haemocytometer based trypan blue cell counting technique [15]. RAW264.7 cells at the density of 1 × 10^6^ cells/mL were seeded in 96-well tissue culture plates and allow to adhere for 2 h. After washing the cells with PBS (pH 7.2), they were allowed to react with the medium mixed with various concentrations of the extracts (0.25–5 mg/mL) followed by 24 h incubation. 24 h incubation time was examined to be the stage that cells remain in the vigorous stage, then suitable to determine the effect on viability. Cell viability was checked after 24 h by removing 100 µL of supernatant and 10 µL of MTT solution was added. After 2 h, the MTT solution was removed, and 100 µL of 0.04 N·HCl in isopropanol was added to dissolve the formazan crystals. Absorbances were recorded at 570 nm using a microplate reader. The percentage of cell viability was calculated by the following Equation (1):

% Cell viability = [Absorbance of sample/Absorbance of control] × 100(1)

### 2.8. Preparation of Endogenous Cellular Extracts

RAW264.7 cells at the density of 1 × 10^6^ cells/mL were seeded in 60 mm tissue culture plates and incubated for 2 h to allow the cells to adhere to the tissue culture plates. Cells were washed with 2 mL of PBS (pH 7.2) before being treated with 6 mL of the extracts at the concentration of 250 and 1000 µg/mL for 24 h. The endogenous cellular fluid was extracted according to a method modified from Du, et al. [16]. Supernatants were removed; cells were washed with PBS and harvested with 0.5 mL of 0.25% trypsin-EDTA. Then, 1 mL of the culture medium was added to stop the reaction, followed by centrifugation at 1000× *g* for 10 min. Cell pellets were washed with PBS until clean and re-suspended in 1 mL of cold PBS. Cells were lysed using a probe-type sonicator (Vibra-Cell, Sonics and Materials Inc., Newtown, CT, USA) by pulsing at 15 s on and 10 s off for 5 cycles on ice. The cell extracts were centrifuged at 10,000× *g* (4 °C) to discard the cell debris, while supernatants were used for the determination of antioxidant enzymes activity and lipid peroxidation assay.

### 2.9. Determination of Endogenous Antioxidant Enzyme Activity

Total superoxide dismutase (SOD) assay was performed using a mixture of 20 μM riboflavin (0.3 mL), 130 mM methionine (0.3 mL), and 100 μM di-sodium ethylenediaminetetraacetic acid (EDTA-Na^2^) (0.3 mL), 50 mM potassium phosphate buffer at pH 7.8 (1.5 mL), deionized water (0.25 mL), 750 μmol/L NBT (0.3 mL), and followed by 0.05 mL of the endogenous cellular extracts. The mixture was placed under light at a photon flux density of 78 μmol photons s^−1^ m^−2^ for 20 min. The absorbance was measured under UV light at 560 nm [17]. The level of glutathione peroxidase (GPx) activity which was the main selenoenzyme involved in antioxidant protection system was measured [18]. The mixture of 0.2 mL endogenous cellular extracts, 0.4 mL of 0.1 mM reduced l-glutathione (GSH) and 0.2 mL of 0.067 M KNaHPO_4_ was pre-heated at 25 °C for 5 min. Afterward, 0.2 mL of 1.3 mM of H_2_O_2_ was added to initiate the reaction and left for 10 min. 1 mL of 1% of trichloroacetic acid was added to terminate the reaction followed by incubation on an ice bath for 30 min. The mixture was centrifuged at 1000× *g* for 10 min and the supernatant (0.48 mL) was then mixed with 0.32 M of Na_2_HPO_4_ (2.2 mL) and 1 mM of DTNB (0.32 mL) in the cuvette. The absorbance at 412 nm was measured under UV light after 5 min (E = 39.4 mM^−1^cm^−1^). Catalase (CAT) assay was investigated from the mixture of 0.3% H_2_O_2_ in 50 mM potassium phosphate buffer pH 7.0 (1.9 mL) with the endogenous cellular extracts (0.1 mL) in the cuvette. Activity of CAT was measured from absorbance change in 60 s at 240 nm (E = 39.4 mM^−1^cm^−1^) [19]. All enzyme activities were calculated according to protein content expressed from the calibration curve of BSA as a standard and expressed as a percentage of the control. The assay of Bradford [20] was used to determined protein content. Reagent was made by dissolving 100 mg of Coomassie brilliant blue G-250 dye in 50 mL of 95% ethanol. The solution was mixed with 100 mL of 85% phosphoric acid and made up to 1 L with distilled water. The reagent was filtered, and 5 mL was added to 0.1 mL of endogenous cellular extract. Absorbance at 595 nm was measured.

### 2.10. Determination of Lipid Peroxidation

Lipid peroxidation assay was measured using thiobarbituric acid reactive substances assay (TBARS) [21]. 1 mL of endogenous cellular extracts was mixed with 4 mL of 20% TCA containing 0.8% of TBA (*w/v*). The mixtures were heated at 95 °C for 60 min, then cooled on ice and centrifuged at 3000× *g* for 10 min. The absorbance was measured at 532 nm. The amount of red complexes was compared to an external standard of MDA. The amount of TBARS was expressed as nmol MDA/mg protein.

### 2.11. Nitric Oxide Synthesis Inhibition Determination

Nitric oxide (NO) synthesis inhibition determination was measured as the screening method for anti-inflammatory properties of ricegrass juice extracts. Briefly, RAW 264.7 cells at the density of 1 × 10^6^ cells/mL were seeded into 96-well plates and allowed to adhere for 2 h. Cells were induced to produce NO by treating them with 100 µL of 100 µg/mL lipopolysaccharide (LPS) from *E. coli* as a foreign matter. 100 µL of extracts at various concentrations from 0.1–1 mg/mL were further added and incubated for 24 h. L-nitro arginine was used as a positive control. Sodium selenite and Se-methionine (Se-Met) from Se-yeast were also used as the sample control. The percentage of NO synthesis inhibition was determined by measuring the accumulation of nitrite in the culture supernatant using the Griess reagent. The supernatant (100 µL) was transferred to 96-well tissue culture plates and then added with 100 µL of Griess reagent. The mixtures were measured using a microplate reader at 570 nm. The percentage of NO synthesis inhibition (%) was calculated from Equation (2). The rest of cells in 96-well plates were rechecked for the number of cell viability with MTT assay to confirm the effectiveness of the extract on the inhibition of NO synthesis.

(2) $$\%\;\mathrm{Inhibition}\;\mathrm{of}\;\mathrm{NO}\;\mathrm{synthesis}=\frac{\left[\left(\mathrm{Control}-\mathrm{Blank}\;\mathrm{of}\;\mathrm{control}\right)-\left(\mathrm{Sample}-\mathrm{Blank}\;\mathrm{of}\;\mathrm{sample}\right)\right]}{\left(\mathrm{Control}-\mathrm{Blank}\;\mathrm{of}\;\mathrm{control}\right)}\times 100$$

### 2.12. Statistical Analysis

All data were expressed as means (*n* = 3) ± standard deviation (S.D.). Comparison between groups was carried out by one-way analysis of variance using SPSS (SPSS Inc., Chicago, IL, USA). Duncan’s test was used to determine significant differences (*p* < 0.05). 

## 3. Results and Discussion

### 3.1. Changes in Se Content and Total Polyphenol Content

**Figure 1:**
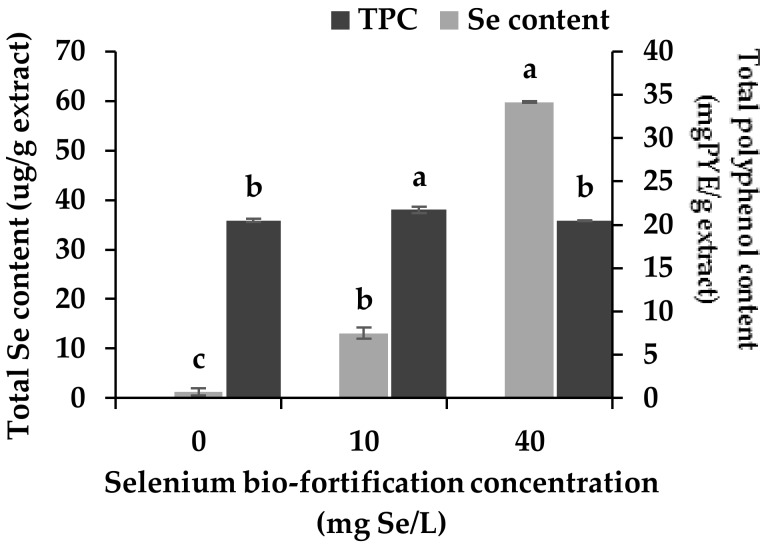
Total selenium (Se) content (µg/g extract) (left axis) and total polyphenol content (right axis) of ricegrass juice extract after being bio-fortified with various concentrations of sodium selenite (0, 10 and 40 mg Se/L) during plantation. Each treatment was tested in triplicate. Data are means ± standard deviation (SD). Different letters indicated significant differences (*p* < 0.05) in Duncan’s significant differences test. PYE: pyrogallol equivalent.

Rice is a plant in the Poaceae family and has been classified as a moderate selenium (Se) accumulating plant. This study aimed to bio-fortify Se in young rice plants, which can exert extra biological properties in combination with polyphenols compounds in ricegrass. According to the preliminary results, however, ricegrass was revealed to have the limitation of not accumulating Se compounds more than 40 mg Se/L, since higher levels of Se concentration led to toxicity, which resulted in significant growth limitation. The addition of Se at 10 and 40 mg Se/L revealed no significant changes in the physiology and yield of ricegrass collected on the day of harvesting [22]. In the present experiment, Se compounds were found to be accumulated in ricegrass in a dose-dependent manner. The supplementation of sodium selenite compounds in the plantation at 10 and 40 mg Se/L resulted in higher levels of Se accumulation than when feeding with water alone. After the aqueous extraction of ricegrass, the highest level of Se compounds that would be achieved in ricegrass juice extract was at the level of 59.76 ± 1.52 µg/g extract at treatment 40 mg Se/L supplementation (Figure 1).

In previous research work, the technique of Se supplementation into plants had been proposed as an alternative path to trigger some of the phytochemical constituents in numerous plants [10]. For example, an enhancement of naringin chalcone and kaempferol level in tomato fruits [23], and a promotion of catechin accumulation in Assam tea has been observed [24]. However, the supplementation of Se compounds into ricegrass during plantation in this study showed a minor modification in the total polyphenol content (TPC) of its aqueous extract. The addition of 10 mg Se/m^3^ led to slightly higher accumulative in TPC level. This may result from a stimulating effect of Se compounds on phenylalanine ammonia lyase (PAL) which is the main enzyme responsible for the synthesis of phenol secondary metabolites in the phenylpropanoids pathway [22,25]. However, plants return to their homeostasis when higher levels were supplemented (treatment 40 mg Se/m^3^).

### 3.2. Phenolic Profiles Identification Using UHPLC-DAD-ESI-MS

In this study, the supplementation of Se compounds into ricegrass appeared to have minimal effects on both the number of phenolic compounds as reported by TPC level and the quality of phenolic compounds. The types of phenolic compounds detected in ricegrass juice extract (RG0) and Se-ricegrass juice extract (RG10, RG40) investigated using HPLC were similar (data not shown). Therefore, ricegrass juice extract (control) was chosen as a representative to identify the main type of phenolic compounds which can be a major substance exhibited the bioactivities using the reversed-phase UHPLC-DAD-ESI-MS, since there was only limited data on the specific types of phenolic compounds found in the aqueous extract of young ricegrass or rice seedlings. The compounds in the present extract were identified based on the UV spectra and a mass ion of each peak was compared to the existing literature.

**Figure 2:**
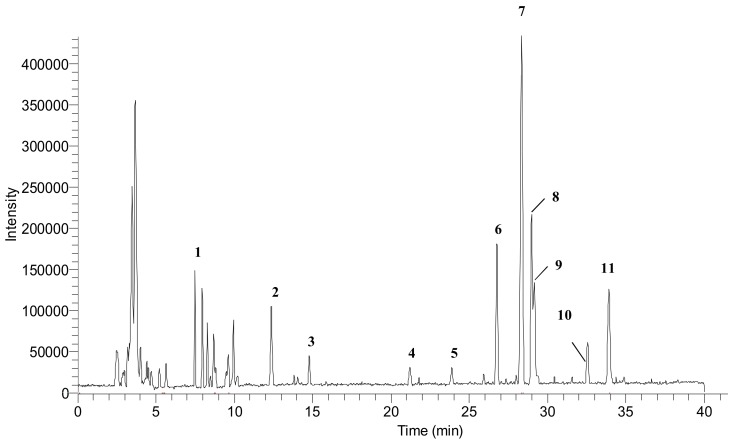
Reversed-phase Ultra High-Performance Liquid Chromatography with diode array detection (UHPLC-DAD) chromatogram of Thai ricegrass juice extract (*Oryza sativa*., Chainat1) with identified peak (1–11).

**Figure 3:**
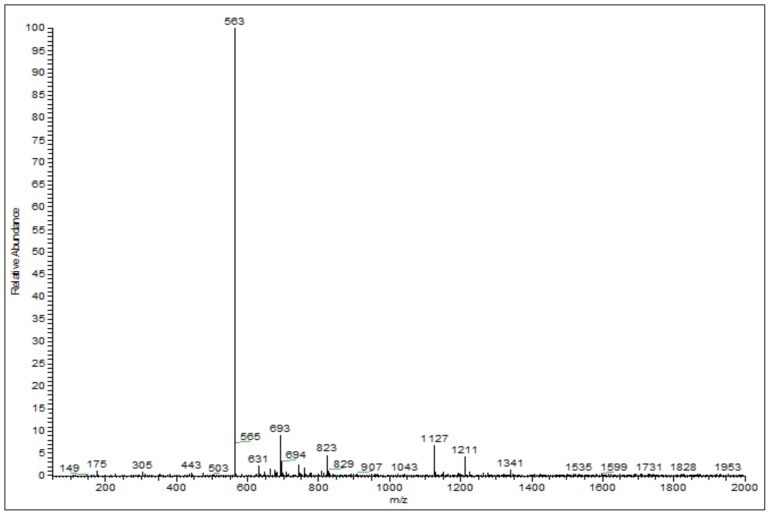
Electro-spray ionization mass spectrum on negative ion of peak 7 (RT 28.71) in ricegrass juice extract.

Figure 2 presents the reversed-phase UHPLC-DAD chromatogram of ricegrass juice extract cultivar Chainat 1 obtained using the suitable gradient elution program. In total, 11 compounds were tentatively identified through the ESI-MS in negative mode considering the UV and MS spectral data. Data concerning the identification of the peaks are shown in Table 1, where the retention time, molecular weight and electrospray ionization mass spectrometry of all the compounds detected are also reported. Peak 1 was observed to represent a base molecular anion at *m*/*z* 191, thus it was identified as quinic acid. Peaks 3 and 4 were identified as phenolic glycosides according to the *m*/*z* of base ions at 315 and 385 resembling the spectrum of protocatechuic glucoside and 1-*o*-sinapoyl-β-d-glucose, respectively. The ion found at the retention time 23.87 min (peak 5), with the *m*/*z* of 367, presented a fragment at *m*/*z* 193 which resembles the *m*/*z* of the ferulic acid fragment. Thus, this compound was consistent with 3-*o*-feruloyl-quinic acid. The large group of secondary metabolite compounds in the leaves of *Oryza sativa* have been reported earlier to be flavone glycosides [26]. The spectra of peaks 6–9 showed a similar base peak of 563 which resembles the spectrum of chrysoeriol arabinosyl arabinoside derivatives. This compound has been formerly found in the literature investigating the compounds in rice leaves using MS/MS with the same *m*/*z* recorded [27]. Peak 7 was recognized as the main compound in ricegrass juice extract. The ESI-MS on negative ion of this peak is presented in Figure 3. Peaks 2, 10 and 11 were the group of compounds which also tentatively identified as flavone glycosides referred to the literature. The *m*/*z* of 325, 445, 491 were detected and they were identified as tricin, swertisin, and tricin-7-*o*-β-d-glucopyranoside, respectively, which are the compounds existing in the leaves of rice and wheat [27,28,29]. 

### 3.3. Cell Viability and Cell Proliferation

The safety aspect was stated as it is necessary for introducing each functional food product into the market. Reliable evidence is needed to make a claim on the cytotoxicity of the extract on mammalian cells. The cytotoxicity of the extracts on RAW264.7, a murine macrophage cell, was chosen since it can represent the influence of compounds specifically on an essential immune system, which is the first defense step in the human body. Macrophages are phagocytic cells which play a crucial role in the clearance of microorganisms, pathogens, and harmful disturbances. Thus, it is also important for the function of antigen presentation, cytokine production and anti-tumor activity [34]. The experiment measured the number of viable cells and cell proliferation after incubating the healthy macrophage cells with the extracts for 24 h.

**Figure 4:**
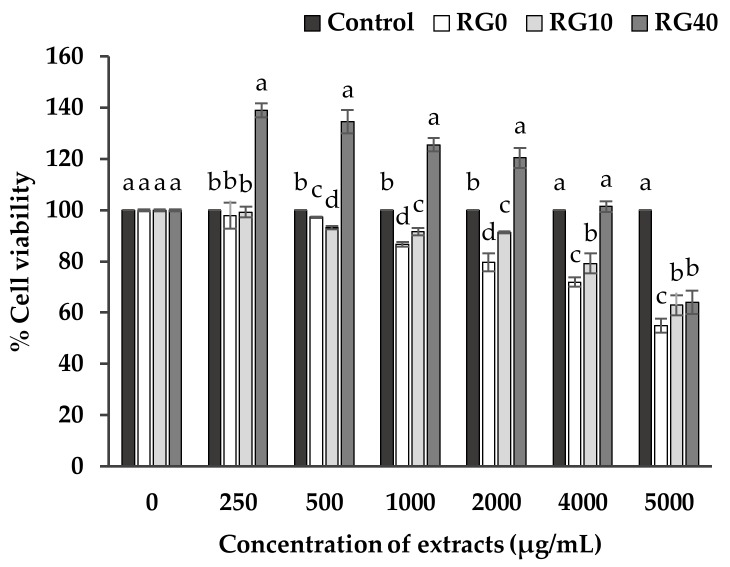
Cell viability and cell proliferation of RAW264.7 murine macrophage cells after incubating with various concentrations of extracts from 250–5000 µg/mL for 24 h: RG0; ricegrass juice extract (control), RG10; ricegrass juice fortified with 10 mg Se/m^3^ extract, RG40; ricegrass juice fortified with 40 mg Se/m^3^ extract. Data are means ± standard deviation (SD). Different letters indicated significant differences (*p* < 0.05) in Duncan’s significant differences test.

Figure 4 reveals the number of cell viability and cell proliferation of RAW264.7 cells after incubating with various concentrations of three extracts (RG0, RG10, RG40), ranging from 250–5000 µg/mL compared to the control. The macrophage cell number slightly decreased through an increased concentration of extracts because an excessive concentration of phenolic compounds, as well as Se compounds in the extract, can act as pro-oxidant molecules which cause damage to cells and lead to cell death. However, the results still confirmed that all of the extracts had no or low toxicity to the RAW264.7 macrophage cells. According to the system of drug screening for neglected diseases, the 50% cytotoxicity concentration (CC_50_) of any substance which was higher than 90 µg/mL was classified as no toxicity [35], thus the extracts were confidently claimed as safe when displaying CC_50_ over 5000 µg/mL. Furthermore, it was found that RG40 greatly promoted the proliferation of RAW264.7 cells (*p* < 0.05) compared to the control especially at low concentration, while RG0 and RG10 did not. All the extracts caused a reduction in the number of the viable cells while the concentration has been increased. The effect of ricegrass juice extract on cell viability in this study can be stated mainly from phenolics and Se. Other literature that checked the effect of Se on mouse monocyte-macrophage cell line (TIB69) also claimed that supplementation of Se influences cell proliferation [36]. Both phenolic and Se compounds have been claimed individually for the positive effects over the cell viability at low amount by being involved as a source of nutrition and possibly playing a role in antioxidant protection in the metabolism of the cells, however, the higher level of both Se and phenolic compounds can cause the oxidative damage to cells and result in cell death. It could be assumed that RG40 at low concentration (250 µg/mL) is the most suitable condition for RAW264.7 cells to grow while RG10 at about 1000 or 2000 µg/mL, though provide similar level of Se compounds to RG40 at 250 µg/mL, it provides higher level of phenolic compounds and may indicate in the reduction in cell viability. Moreover, the bio-fortification of Se into ricegrass may propose changes in various components not only phenolic compounds and selenium content. The LCMS analysis may only visible some compounds contained in the extracts which do not differ from each other. The other compounds may play some role in the cell proliferation as well as cell reduction.

### 3.4. Lipid Peroxidation and Antioxidant Enzymes Activity

Oxygen is the primary agent used for the respiration and the metabolism of living organisms. ROS are a natural byproduct of the normal metabolism of oxygen. Generally, it has had an important role in cellular signaling and defense against pathogens [37]. However, during times of environmental stress, ROS levels can increase dramatically, and the elevated amounts include superoxide anion (O_2_•^−^), hydroperoxyl (perhydroxyl) radical (HO_2_•), hydroxyl radical (•OH), and nitric oxide (NO), resulting in significant damage to cell structures. Living organisms contain lipid as the main structure of cellular membranes [38]. Thus, lipid peroxidation can be stated as a crucial step in the pathogenesis of several disease states in humans such as atherosclerosis, Alzheimer’s disease, and cancer. Therefore, the extent of lipid peroxidation byproducts produced like malondialdehyde (MDA) can reflect the extent of oxidative damage to cells [39].

**Figure 5:**
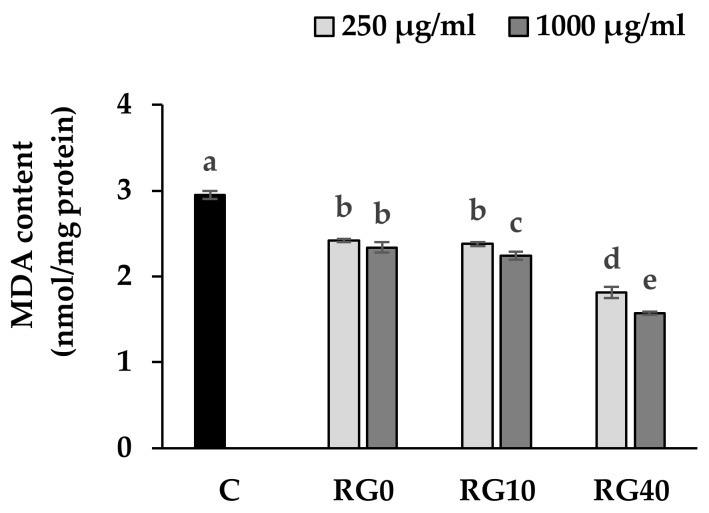
Level of malondialdehyde (MDA) in RAW264.7 cell line treated with selenium biofortified ricegrass juice extract (RG0, RG10, RG40) at level 250 and 1000 µg/mL compared to control. RG0; ricegrass juice extract (control), RG10; ricegrass juice fortified with 10 mg Se/m^3^ extract, RG40; ricegrass juice fortified with 40 mg Se/m^3^ extract. Data are means ± standard deviation (SD). Different letters indicated significant differences (*p* < 0.05) in Duncan’s significant differences test.

The level of MDA detected from the cellular extracts of RAW264.7 cells after treatment with the extracts, therefore, revealed the effect of ricegrass juice extract and Se-enriched ricegrass juice extract on the role of oxidative stress protection (Figure 5). Outcomes indicated that ricegrass juice extracts of all treatments can significantly reduce the level detected of MDA from RAW264.7 cellular compared to the normal cells. The role of phenolic compounds in the extracts was considered as having the major effects. Phenolic compounds possess a mechanism to lower the MDA level through the ability as an electron donor, which can stabilize the hydroxyl radicals and lipid peroxyl radicals, thereby lowering the extent of oxidative damage to the lipid cell membrane. In addition, an increased level of Se content in the RG10 and RG40 showed more reduction in MDA content compared to the RG0 at dose 1000 µg/mL.

In order to clarify the conceivable mechanism of the extracts in lowering the level of lipid peroxidation, their ability to regulate three main endogenous antioxidant enzymes including SOD, CAT, and GPx was also studied. SOD is the defense enzyme that deals with superoxide radicals (O_2_•) which are the first radicals generated from oxygen metabolism and convert them to hydrogen peroxide (H_2_O_2_) which CAT and GPx are occupied and further transformation to harmless water (H_2_O) which terminates the damaging chain reaction [40]. 

**Figure 6:**
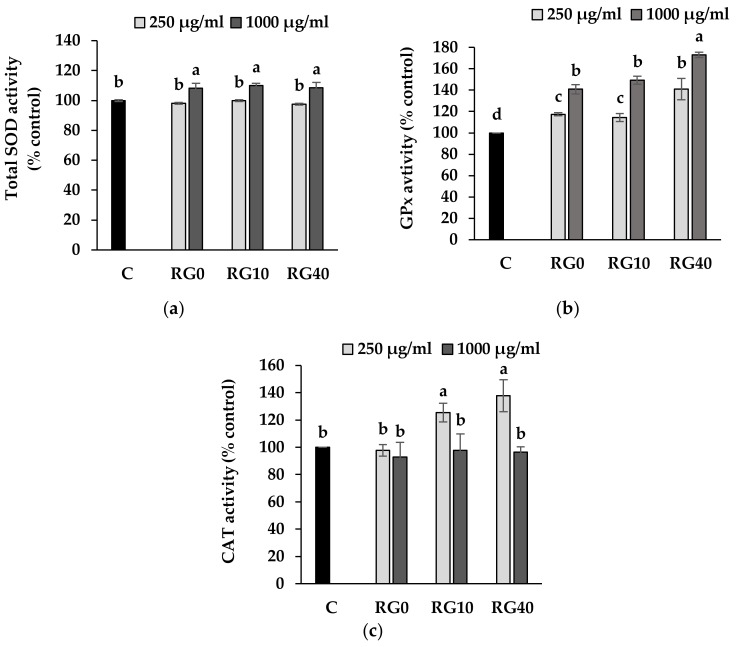
Level of SOD (**a**), GPx (**b**), and CAT (**c**) in RAW264.7 cell line treated with RG0, RG10, and RG40 compare to control. RG0; ricegrass juice extract (control), RG10; ricegrass juice fortified with 10 mg Se/L extract, RG40; ricegrass juice fortified with 40 mg Se/L extract. Data are means ± standard deviation (SD). Different letters indicated significant differences (*p* < 0.05) in Duncan’s significant differences test.

All the extracts at low concentration seemed to have no effect on the level of total SOD. Yet, at a higher concentration, the was a slight increase total SOD. No effect was detected from the different concentration of Se in the extracts (Figure 6a). The addition of extracts to RAW264.7 cells in all treatments was able to promote the activity of GPx which are classified as the seleno-enzymes. It appeared that the RG40 at 1000 µg/mL can increase the highest proportion of GPX activity, up to 73% above the control (Figure 6b). Since Se is the main cofactor of this enzyme, extracts that contain a high level of Se can then logically promote a greater level of GPx activity. In the CAT assay, low level of the extracts at RG10 and RG40 treatment showed an increasing CAT activity above the level of normal cells (C), while the level of CAT in RG0 showed no change. However, a higher level of the extracts at 1000 µg/mL suppressed CAT activity (Figure 6c). This phenomenon can be explained by the fact that GPx and CAT had grossing similar roles in converting H_2_O_2_ to H_2_O. While GPx was high enough to reduce the level of the substrate, the CAT was probably sparing in the cells to remain in homeostasis. According to the results, ricegrass juice extracts revealed the supportive activity on all three antioxidant enzymes above the regular one. Animal studies on the effect of dietary supplementation of phenolic compounds on antioxidant enzymes levels in rat suggested that phenolic compounds selectively induced the mRNA expression through the upregulation of gene transcription and Nrf2 transcription factor [41]. Moreover, it was noticed that the tested extracts provided larger effects on CAT and GPx enzymes compared to total SOD. The reduction in MDA content from the cellular extracts of cells treated with the test samples especially in RG40 treatment, thus indicating that the proposed role of these two enzymes is via both Se element and phenolics functions.

### 3.5. Nitric Oxide Inhibition Properties

**Figure 7:**
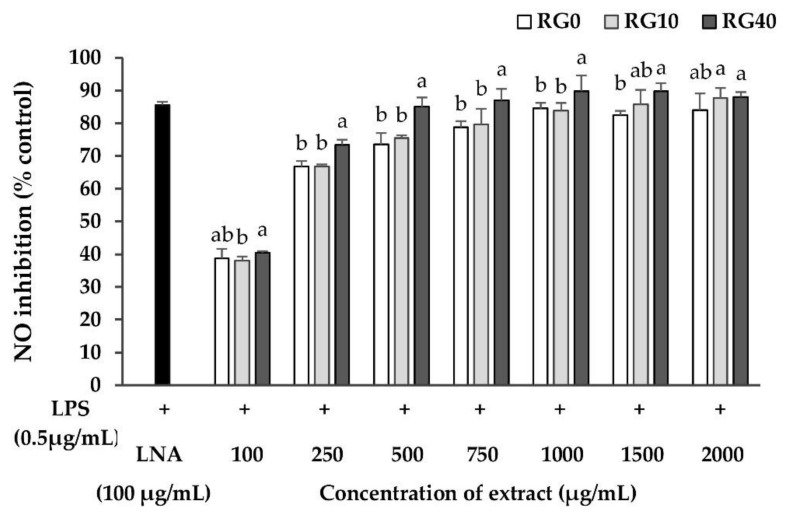
The effect of extracts on the inhibition of nitric oxide (NO) in RAW264.7 murine macrophage cells after co-incubation of LPS from *E. coli* (0.5 µg/mL) with various concentrations of extracts from 100–1000 µg/mL for 24 h using LNA as a positive control (100 µg/mL): RG0; ricegrass juice extract (control), RG10; ricegrass juice fortified with 10 mg Se/L extract, RG40; ricegrass juice fortified with 40 mg Se/L extract. Data are means ± standard deviation (SD). Different letters indicate significant differences (*p* < 0.05) in Duncan’s significant differences test.

Nitric oxide (NO) is a major mediator produced by macrophages during inflammation responses. It is produced by the enzyme inducible nitric oxide synthase (iNOS) from the substrate, arginine [42]. NO is vital for many physiological functions such as being a neurotransmitter and affecting blood flow and synaptic plasticity. On the other hand, an excess amount of NO can destroy and induce dysfunction in the macrophages themselves as well as in surrounding normal cells [43]. Therefore, the study on plant foods that can modulate the level of NO produced by its homeostasis is an important issue because they are safer, have limited side effects, and are relatively low cost compared with medication. In this study, LPS from *E. coli* as a foreign matter was applied to RAW264.7 cells to initiate the production of NO. Then, RG0, RG10, and RG40 were co-treated to analyze their NO production inhibitory effects. The concentration of ricegrass juice extracts which did not affect the percentage of cell viability to be lower than 80% was 2000 µg/mL. Thus, this range was the maximum concentration used for the determination of nitric oxide inhibition properties to verify that NO was truly produced. The level of LPS which did not affect cell viability and could stimulate the highest level of NO was scanned and the level of LPS at 0.5 µg/mL was chosen. The percentage of NO production inhibition was measured using the Griess assay and it was shown as the percentage of negative control (Figure 7). The co-incubation experiment of LPS and the extracts at various concentration showed a dose-dependent inhibition of NO production. Results revealed that NO from LPS-induced RAW264.7 was significantly inhibited by ricegrass juice extracts. The Se biofortified one, RG40, showed the greatest ability to inhibit NO compared to other treatments. The IC_50_ of RG0, RG10, and RG40 were 147.02, 147.73, and 118.76 µg/mL, respectively. Although the IC_50_ values were still higher compared to the positive control, L-nitro-arginine (LNA), which is known as the enzyme iNOS inhibitor (IC_50_ = 30.05 µg/mL). Therefore, ricegrass juice in all treatments was still on a considerable amount that can be classified as functional food.

The NO inhibition of ricegrass juice extract can be initially attributed to the role of phenolic compounds. A correlation between high intake of phenolic compound rich food and the ability to downregulate the inflammatory responses in vitro and in vivo has been previously reported [44,45]. It has been hypothesized that phenolic compounds exert anti-inflammatory activity by inhibiting the synthesis of pro-inflammatory mediators, modification of eicosanoid synthesis, inhibition of activated immune cells, or inhibition of iNOS and cyclooxygenase-2 via inhibitory effects on nuclear factor NF-κβ [46]. Main components of phenolic compounds found in ricegrass juice extract were in the group of flavone glycosides. The previous study has reported on the effect of flavonoid compounds like apigenin and quercetin on NO; the results indicated that the inhibition of NO was due to the reduction of iNOS expression [47]. Moreover, flavone compounds may possess scavenging ability since NO is one of the reactive oxygen species (ROS) that is a by-product formed during the mitochondrial respiratory process [41]. Phenolic compounds can effectively scavenge the radicals as well as NO radical through the antioxidation process by stabilizing the radicals through the active site of -OH group and the planar ring. Thus, a reduction in the NO level in cell surrounding was found. 

**Figure 8:**
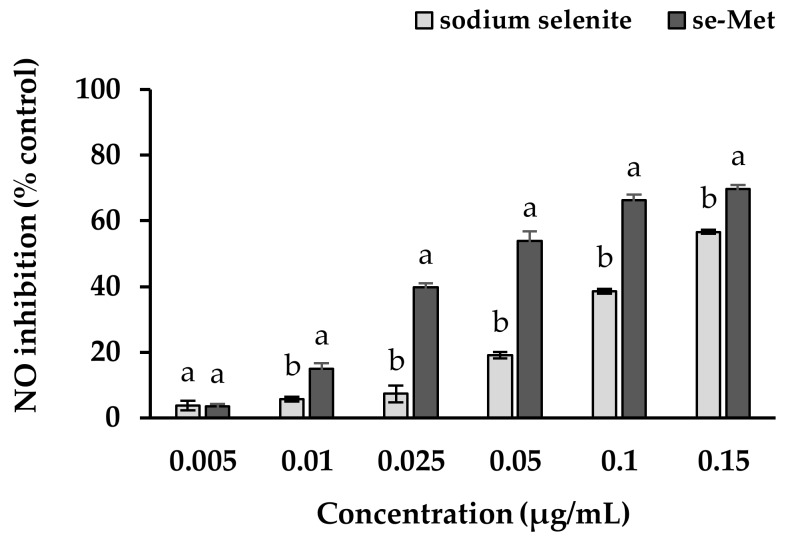
The effect of extracts on the inhibition of nitric oxide (NO) in RAW264.7 murine macrophage cells after co-incubation of LPS from *E. coli* (0.5 µg/mL) with sodium selenite, seleno-methionine (se-Met) from Se-yeast for 24 h. Data are means ± standard deviation (SD). Different letters indicate significant differences (*p* < 0.05) in Duncan’s significant differences test.

Another segment that influences the reduction of NO production may relate to the activity of Se. Since RG40 exerted approximately an extra 5% on NO inhibition activity compared to RG0 and RG10, the effect of Se itself on NO inhibition was then studied to confirm the additional benefit (Figure 8). The experiment was designed to use two forms of Se compounds including sodium selenite as inorganic Se supplement and seleno-methionine (Se-Met) from Se yeast as organic Se supplement. Se in both forms exerted NO inhibition properties at a low dose, which less than 0.15 µg/mL, while a higher dose of the pure compounds was toxic to the cells. Furthermore, Se-Met exerted higher ability than sodium selenite to reduce the level of NO. The underlying mechanisms of Se to inhibit the production of NO might be that Se as an essential part of the enzyme glutathione peroxidase (GPx), Se-GPx active site may exert its chemo-preventive effect by inhibiting the expression of iNOS and subsequently inhibiting NO production [48]. Moreover, the organic form of Se provided higher ability could be due to the bioavailability of the organic Se in which the element was already bound to amino acid and could be utilized straightforwardly as a part of the enzyme while inorganic form like selenite needs to initially be transformed to the organic form before being utilized, so an extra stage is required [49]. Since the majority of Se in the ricegrass juice extract was supposed to be an organic form, the RG40 treatment that contained five times of Se content than the control may provide more advantages from the higher level of organic Se. These outcomes provided evidence for the beneficial effects of dietary Se supplementation in the prevention and or treatment of oxidative-stress-mediated inflammatory diseases.

The experiment was designed to test the effect of the ricegrass juice extract on antioxidant activities in the non-stimulating model because it represented the normal condition of healthy people because we would like to examine whether the consumption of ricegrass juice can promote the antioxidant defense system in a normal human body, since the oxidative damage naturally occurred all the time even in the normal condition. However, the inflammation is the more complex process which did not occur naturally. Thus, the induction of nitric oxide production using foreign matter (LPS) is required to examine this property. However, future work could also further examine the effect of the extracts on stimulating condition like H_2_O_2_ oxidative stress or Cd stress induction. 

## 4. Conclusions

Se-rich ricegrass juice extract can be classified as safe since it had no toxicity on RAW264.7 murine macrophage cells. Additionally, RG40 promoted proliferation of the cells. Flavone glycosides were identified as the main phenolic compounds in ricegrass juice extracts and possessed biological properties on the reduction of oxidative stress and NO inhibition. This finding was the first work to report the ability of ricegrass juice to play a part in the treatment of anti-inflammation. Moreover, Se supplemented treatment (RG40) reduced the greatest level of MDA content and inhibited NO production in which the mechanism was related to an increase in CAT and GPx activities. Therefore, Se-rich ricegrass juice can be considered for production as a value-added functional drink from rice that can lower risk of oxidative stress and chronic inflammatory disease and promote human well-being.

## Figures and Tables

**Table 1:** Tentative identification of phenolic compounds in ricegrass juice extract analyzed by Ultra High-Performance Liquid Chromatography with electrospray ionization-mass spectrometry (UHPLC-ESI-MS).

Peak No. ^a^	RT (min)	Tentative Compounds	MW	[M − H] + (*m/z*)	Fragment Ion	Ref.
1	7.95	Quinic acid	192	191	111, 192, 613	[30]
2	12.38	Tricin	330	329	175, 461	[27,28]
3	14.79	Protocatechuic glucoside	316	315	175, 445, 575	[31,32]
4	21.20	1-*o*-Sinapoyl-β-d-glucose	386	385	175, 469, 599	[27]
5	23.87	3-*o*-Feruloylquinic acid	368	367	193, 435, 497	[27,33]
6	26.75	Chrysoeriol arabinosyl arabinoside derivatives	564	563	175, 305, 693	[26,27]
7	28.31	Chrysoeriol arabinosyl arabinoside derivatives	564	563	565, 693	[26,27]
8	29.00	Chrysoeriol arabinosyl arabinoside derivatives	564	563	693	[26,27]
9	29.14	Chrysoeriol arabinosyl arabinoside derivatives	564	563	693	[26,27]
10	32.56	Swertisin	446	445	175, 305	[27]
11	33.90	Tricin-7-*o*-β-d-glucopyranoside	492	491	769, 983	[27]

^a^ Peak numbers and retention times refer to Figure 1. RT: retention time. MW: Molecular weight.
